# Culturally adapted intervention regarding decision‐making on feeding options for Chinese American dementia caregivers: A human‐centered approach

**DOI:** 10.1002/alz.088317

**Published:** 2025-01-09

**Authors:** Yaolin Pei, Jing Wang, Dena Schulman‐Green, Terri Fried, Laura Hanson, Bei Wu

**Affiliations:** ^1^ New York University, New York, NY USA; ^2^ 4 Library Way, Durham, NH USA; ^3^ Yale School of Medicine, New Haven, CT USA; ^4^ The University of North Carolina at Chapel Hill, Chapel Hill, NC USA

## Abstract

**Background:**

Studies show that tube feeding does not improve clinical outcomes, and professional guidelines recommend against its use for individuals with advanced dementia. Yet, our preliminary work demonstrates a preference for tube feeding among Chinese‐American dementia caregivers. We propose linguistic and cultural adaptation of “Making Choices: Feeding Options for Patients with Dementia (MCFODA) to create the Chinese version of this efficacious decision aid intervention. The study aims to inform the process and domains of the cultural adaptation.

**Method:**

The first step of the adaptation process is interviews with Chinese‐American dementia caregivers to learn about their decision‐making experiences around feeding options. To identify participants, we collaborated with Caringkind, an agency of servicing dementia caregivers, and used purposive sampling. We began conducting semi‐structured interviews via Zoom with a target sample of 25. Participants completed a brief demographic questionnaire. Audio recordings were transcribed in Chinese and translated into English by a multilingual research assistant. Thematic analysis was used in this study.

**Result:**

Caregivers reported various strategies to prevent choking and ensure patients’ healthy eating, including adding thickness, blending food, and making soup. Caregivers and other family members participated in decision‐making regarding feeding options. To maintain family harmony, timely communication about patients’ health status is one of the important strategies they used for the family decision‐making about feeding options. Factors affecting family decision‐making of whether to use tube feeding included the PLWD’s health status, other people’s end‐of‐life experience of using tube feeding, PLWD’s preferences toward tube feeding and personality, the cultural importance of food, and caregivers’ perception of comfort in feeding options. PLWD’s wishes were perceived as important in decision‐making. However, family were often unaware of these wishes, as they had not been discussed, although some PLWDs expressed their wishes indirectly by discussing other people’s experience in movies or TV drama. Chinese translators were reported as critical during discussions between Chinese families and health care providers.

**Conclusion:**

Engaging end‐users in the adaptation of MCFODA will optimize development of the culturally adapted intervention. For Chinese Americans, we plan to summarize caregivers’ practices used to manage PLWD’s eating, provide suggestions for good family communication, and how to identify PLWD’s wishes.

